# Evaluating the Patterns of Maize Development in the Hetao Irrigation Region Using the Sentinel-1 GRD SAR Bipolar Descriptor

**DOI:** 10.3390/s24216864

**Published:** 2024-10-25

**Authors:** Hexiang Zheng, Hongfei Hou, Delong Tian, Changfu Tong, Ziyuan Qin

**Affiliations:** Institute of Water Resources for Pastoral Area Ministry of Water Resources, Hohhot 010020, China

**Keywords:** SAR, Hetao irrigation area, unsupervised clustering framework, maize

## Abstract

Assessing maize yield is critical, as it is directly influenced by the crop’s growth conditions. Therefore, real-time monitoring of maize growth is necessary. Regular monitoring of maize growth indicators is essential for optimizing irrigation management and evaluating agricultural yield. However, quantifying the physical aspects of regional crop development using time-series data is a challenging task. This research was conducted at the Dengkou Experimental Station in the Hetao irrigation area, Northwest China, to develop a monitoring tool for regional maize growth parameters. The tool aimed to establish a correlation between satellite-based physical data and actual crop growth on the ground. This study utilized dual-polarization Sentinel-1A GRD SAR data, accessible via the Google Earth Engine (GEE) cloud platform. Three polarization descriptors were introduced: *θ*_c_ (pseudo-scattering type parameter), *H*_c_ (pseudo-scattering entropy parameter), and *m*_c_ (co-polar purity parameter). Using an unsupervised clustering framework, the maize-growing area was classified into several scattering mechanism groups, and the growth characteristics of the maize crop were analyzed. The results showed that throughout the maize development cycle, the parameters *θ*_c_, *H*_c_, and *m*_c_ varied within the ranges of 26.82° to 42.13°, 0.48 to 0.89, and 0.32 to 0.85, respectively. During the leaf development stage, approximately 80% of the maize sampling points were concentrated in the low-to-moderate entropy scattering zone. As the plants reached the big trumpet stage, the entire cluster shifted to the high-entropy vegetation scattering zone. Finally, at maturity, over 60% of the sampling points were located in the high-entropy distribution scattering zone. This study presents an advanced analytical tool for crop management and yield estimation by utilizing precise and high-resolution spatial and temporal data on crop growth dynamics. The tool enhances the accuracy of crop growth management across different spatial and temporal conditions.

## 1. Introduction

SAR data are very responsive to the structure of crop canopies and their dielectric characteristics [1]. As a result, SAR data have been extensively used for monitoring and categorizing crop growth, estimating crop yields, and characterizing different phases of plant development [2]. SAR is crucial for continuously monitoring changes in agricultural phenology, particularly at the field level and over extended periods [3]. It serves as a valuable instrument for tracking the dynamics of crop development.

The use of dual-polarization SAR data acquired from the Sentinel-1 satellite in the GEE platform offers a significant approach for crop monitoring in many applications [4]. The dual-polarization SAR mode sacrifices polarization information in exchange for a wider bandwidth and lower data volume, as compared to the full-pol mode [5]. Given the superior spatial resolution and flexibility to operate in all phenology conditions, dual-polarization SAR data shows great potential for ongoing monitoring at the field level [6]. Time-series satellite imaging may achieve exceptional levels of accuracy in observing maize phenology using LSP (Land Surface Phenology).

Long-term monitoring of agricultural growth characteristics and phenology is a key focus in worldwide change research [7]. Nevertheless, the spatial resolution of LSP may not consistently align with the size of crop changes, and the phenology derived from this approach may not accurately reflect the specific phases seen in the field [8]. Although Landsat has a greater level of detail in terms of spatial resolution, it cannot catch the trends that occur across several years, known as interannual patterns, in LSP [9]. To overcome this limitation, Dunn et al. assessed long-term phenology estimates by combining data from many years to guarantee sufficient observations during the whole growing season [10]. The spatiotemporal image fusion technique combines the benefits of gathering both Landsat and MODIS data [11]. It utilizes the daily frequency of MODIS and the high resolution of Landsat to forecast high-resolution imaging (30 m) in the Landsat observations [12]. Double-pol backscatter intensity has the potential to be used for identifying crop types and estimating crop biophysical characteristics [13]. At present, SAR data is extensively utilized in clustering techniques for characterization [7]. A clustering technique called dual-polarization (VV-VH) is suggested, which involves decomposing a 2 × 2 covariance matrix to characterize the scattering mechanism [14]. The scattering angle *α* is determined by the pseudo-probability weighting associated with the eigenvalues of the two orthogonally polarized states [15]. Jiao et al. colleagues use the eigendecomposition approach to add a scattering-type parameter, denoted as *θ*, for the dual-polarization SLC data (HH-VH) [16]. However, it is important to note that this parameter carries distinct scattering information compared to *α* [17]. This study utilizes an image fusion technique to estimate the time series LSP. Additionally, an unsupervised clustering framework is implemented, based on the scattering mechanism, using *θ* and the scattering entropy (H) to partition the non-clustered plane into four distinct regions based on the scattering type. The approach put out by Touzi et al. attains polarization basis invariance by diagonalizing the Kennaugh-Huynen scattering matrix and projecting it into the Pauli basis, hence enhancing the stability of SAR data across various polarization states [18]. This is crucial for assessing the polarization properties of crop growth, facilitating a more precise evaluation of scattering mechanisms at various growth stages, independent of polarization variations. The scattering power decomposition approach utilizing geodesic distance (GD), introduced by Muhuri et al., mitigates pseudo-power through CpRVI, hence diminishing errors induced by target imperfections in agricultural terrains [19]. This compensatory technique enhances the precision of scattering power in dual-polarization SAR data, thereby advancing the comprehension of scattering mechanisms in agricultural regions and yielding more accurate assessments of biophysical parameters.

In addition, the crop monitoring and retrieval of biophysical parameters included the use of three different types of radar vegetation indices: dual-polarization radar vegetation index (RVI), dual-polarization SAR vegetation index (DPSVI), and dual-polarization radar vegetation index (DpRVI) [20]. However, dual-polarization GRD SAR data packages cannot be described using the same terms [21]. Narayanarao Bhogapurapu et al. introduced three dual-polarization descriptors: *m*_c_, *θ*_c_, and *H*_c_. They also proposed a new unsupervised clustering framework that describes six feasible clustering zones representing different scattering mechanisms [22]. This framework efficiently characterizes different phenological phases and helps in detecting crop errors [23]. The cross-pol ratio is a crucial measure for monitoring crop biophysical parameters and phenology in the time-sensitivity study of crops [24]. Zhang et al. introduced a method to analyze the VV-VH backscattering intensity time trend of winter wheat crops using a flat hexahedral approach [25]. They then used this study to distinguish between distinct phenological stages based on the time trend and slope of VH/VV. Moreover, deep learning-based target monitoring systems possess considerable advantages in plant surveillance. Chen et al. enhanced the YOLO-v4 model and introduced a technique for detecting bayberry trees in high-resolution drone imagery, exhibiting exceptional efficacy in the swift monitoring and enumeration of regional crops [26]. YOLO, as a comprehensive object identification model, employs its robust convolutional neural network architecture to sustain elevated detection accuracy in challenging conditions. Nonetheless, deep learning techniques such as YOLO necessitate substantial training data to effectively identify and categorize crops and their growth stages [27]. Insufficient training data significantly impairs the model’s accuracy, leading to diminished generalization capability. Conversely, unsupervised clustering techniques, which operate independently of data, optical imagery, and climatic factors, possess considerable advantages in regional and temporal crop monitoring.

**Figure 1:**
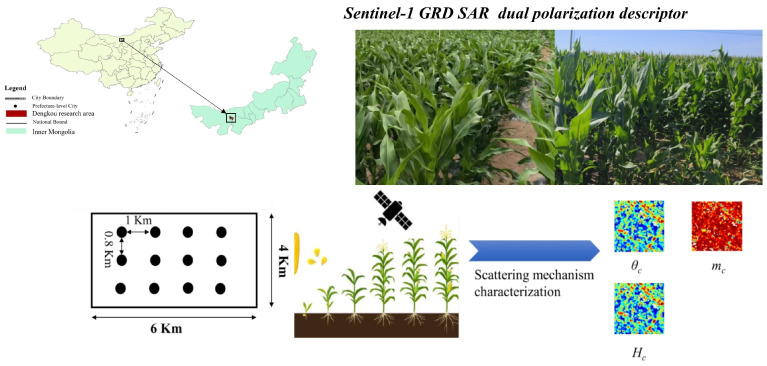
Methodology Flowchart.

This study addresses the escalating global drought stress on crops by employing the GEE cloud platform, utilizing Sentinel-1A GRD SAR data and time-series image fusion technology to create a comprehensive monitoring system for crop growth physical parameters and phenological characteristics. This system seeks to conduct a comprehensive analysis of the temporal growth and development of crops, establishing a robust scientific foundation for enhancing crop yields in the Hetao irrigation area (methodological flow is illustrated in Figure 1). In contrast to single-polarization SAR, our technique acquires additional scattering information, yielding a more comprehensive perspective of surface characteristics. In contrast to the YOLO model, unsupervised clustering obviates the need for extensive optical datasets, hence diminishing data preparation and training expenses—crucial for sustained crop monitoring. Dual-polarization SAR can penetrate clouds, facilitating dependable monitoring in cloudy circumstances and reducing climate-related interruptions in time-series analysis. The primary objectives of this study are: (1) to utilize the Dengkou experimental station in the Hetao irrigation area as the research focus, establishing an unsupervised clustering framework based on Sentinel-1A GRD SAR data to evaluate the physical parameters of corn growth and analyze significant alterations in phenological characteristics; and (2) to further assess the phenological characteristics of corn growth, thereby offering effective scientific instruments for agricultural crop monitoring and sustainable development.

## 2. Materials and Methods

### 2.1. Study Area

**Figure 2:**
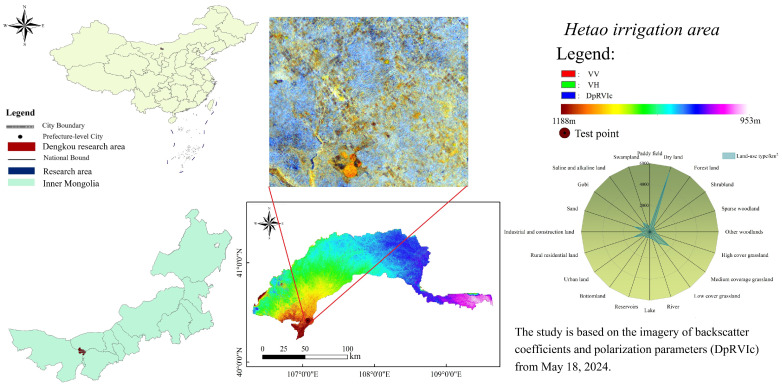
Geographic location of the study area.

The Dengkou test area is situated in the semi-arid and arid Hetao Irrigation Area in northwest China. It is located in Wutian Banner, Ordos City, Inner Mongolia Autonomous Region, between coordinates 108°83′–109°68′ E and 38°32′–39°10′ N. The average elevation of the area is 1316 m, as shown in Figure 2. The test area covers approximately 2986 km^2^ of land and has terrain that is higher in the west and lower in the east. The test region is characterized by a moderate continental monsoon climate, with an average annual temperature ranging from 6.0 to 8.5 °C. The area receives an annual precipitation of 220–400 mm and experiences an annual evaporation of 2100–2600 mm. Additionally, the average annual wind speed in the area is 4.5 m/s [28]. The test location exhibits an arid environment with high evaporation rates, irregular rainfall patterns, intense solar radiation, and windy and sandy conditions. The soil in the research area is classified into two layers: the upper layer (0~40 cm) consists of chalky loam with a soil density of 1.43 g/cm^3^, while the bottom layer (40~100 cm) also consists of chalky loam but with a slightly lower soil density of 1.40 g/cm^3^. The levels of soil organic matter, total nitrogen, effective phosphorus, fast-acting potassium, and slow-acting potassium were measured by the soil test and soil quality guidelines. The results showed concentrations of 8.27 g kg^−1^, 509 mg kg^−1^, 5.5 mg kg^−1^, 5.5 mg kg^−1^, and 5.5 mg kg^−1^, correspondingly. The values are expressed as milligrams per kilogram (mg/kg): 5.3 mg/kg, 183 mg/kg, and 536 mg/kg.

### 2.2. Experimental Design

**Figure 3:**
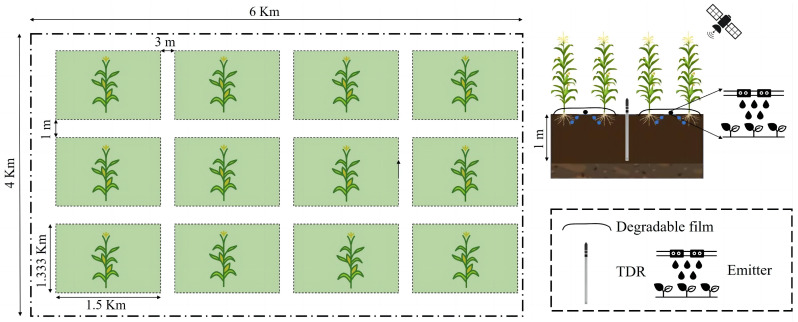
Layout of the test area.

The test location has a 4 km × 6 km maize cultivation area, divided into 12 plots (each measuring 1.333 km × 1.5 km), with 3 m protection strips on the eastern and western peripheries and 1 m on the northern and southern boundaries. Irrigation was overseen by local farmers and ranchers, using Yellow River water with a total dissolved solids content of 0.71 g/L. Maize was planted in mid-April, with spring irrigation performed 10 days before sowing, and harvested by the end of September. The cultivar used was “Shaanxi Single 609”, with high-pressure polyethylene black mulch and a sowing density of around 4000 seeds per acre. The configuration is shown in Figure 3.

### 2.3. Experimental Remote Sensing Monitoring

**Figure 4:**
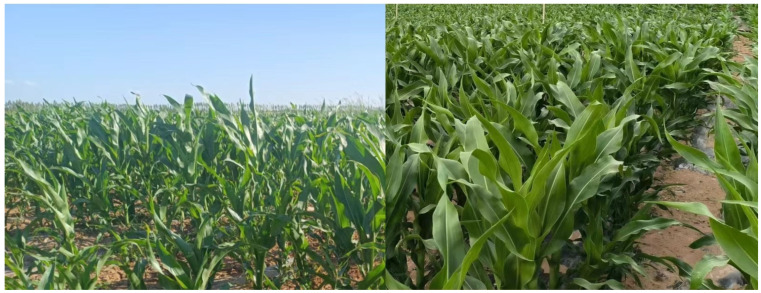
Field photographs of maize at different phenology stages.

This research examined 12 maize fields. Figure 3 and Figure 4 illustrate field photographs at various development phases and the allocation of designated areas. We gathered in-situ data on maize and soil inside the research region and used Sentinel-1’s C-band (central frequency: 5.405 GHz) for data collection. The Interferometric Wide (IW) mode was used, including a swath width of 25 km and a spatial resolution of 5 m by 20 m. The Noise Equivalent Sigma Zero (NESZ) measured −25 dB within an incidence angle range of 20° to 46°. The research used eight dual-polarization (VV-VH) Sentinel-1A GRD SAR pictures, including incidence angles ranging from 30.65° to 41.76°. Comprehensive information on the SAR data is included in Table 1. The data were gathered according to the phenological phases of maize over 13 designated days of the year (DOY-126, 138, 150, 162, 174, 186, 198, 210, 222, 234, 246, 258, 270).

### 2.4. Data and Processing

#### 2.4.1. Meteorological Data

We performed a quantitative analysis of the experimental region utilizing the MOD16A2 evapotranspiration (*ET*) product from the MODIS dataset on the GEE platform. MOD16A2, including an 8-day temporal resolution and a 500-m spatial resolution, was employed in this work to evaluate the evapotranspiration conditions of the experimental area. The examined meteorological data encompasses temperature, precipitation, humidity, solar radiation, atmospheric pressure, and wind velocity. Utilizing this data, we computed the long-term reference evapotranspiration (*ET*_0_) and the yearly average potential evapotranspiration (*PET*) to assess the influence of evapotranspiration on crop development [29].

#### 2.4.2. Soil Moisture Content

In our soil physics research, we used the commonly utilized Time Domain Reflectometry (TDR) technique. The technique for estimating soil moisture is detailed in [30]. We established 36 TDR soil moisture monitoring points covering depths of 0–100 cm, with weekly measurements to capture the main root distribution zone of the crops. This setup aims to comprehensively assess crop water demand and utilization, with TDR probes calibrated using the soil drilling method.

#### 2.4.3. Hydroclimatological Information

The meteorological data for this research is sourced mainly from two platforms: the ERA5 dataset in GEE and the National Tibetan Plateau Data Center of China. The ERA5 dataset offers daily average temperature and precipitation data from 2010 to 2020, derived from the mean_2m_air_temperature and total_precipitation variables, respectively [31].

### 2.5. Research Methodology

#### 2.5.1. Unsupervised Clustering Framework

The omnipresent, continuous monitoring capabilities of Sentinel-1A SAR data make it very appropriate for agricultural applications. Utilizing dual-polarization data mitigates cloud interference difficulties that often impact optical remote sensing data, enabling continuous crop monitoring. This is particularly crucial for the sustained collection of data in agricultural monitoring. This research used an unsupervised clustering framework to observe the development stage of maize using Sentinel-1A GRD SAR data. The growth stage was determined by analyzing the distinct scattering features of the three dual-polarization descriptors. The research may evaluate the scattering processes of surface targets more fully in complicated agricultural landscapes with these polarization descriptors. Furthermore, the unsupervised clustering technique requires less training data, hence diminishing human involvement and enhancing analytical efficiency. The co-polarization purity parameter (*m*_c_) in this research is represented by the symbol *q*, which is calculated using the following equation [32]:

(1) $$m_{c}=\frac{1-q}{1+q};0\leq m_{c}\leq 1$$

where *q* is the ratio parameter of *σ°_XY_/σ°_XX_*.

In this research, the values *σ°_XX_*, *σ°_XY_*, and *m*_c_ are used to define two auxiliary quantities [22]:

(2) $$tan\zeta_{1}=\frac{{\sigma {}^{\circ}}_{XX}}{m_{c}I};\;tan\zeta_{2}=\frac{{\sigma {}^{\circ}}_{XY}}{m_{c}I}$$

where the total intensity, *I* = *σ^∘^_XX_* + *σ^∘^_XY_*, is brought to obtain:

(3) $$tan\theta_{1}=tan\left(\zeta_{1}-\zeta_{2}\right)=\frac{{\left(1-q\right)}^{2}}{1+q^{2}-q};\;0{}^{\circ}\;\leq \;\theta_{c}\;\leq \;45{}^{\circ}$$

The scattering between *m*_c_ and *θ*_c_ has intricately correlated properties. Consequently, we used the pseudo-scattering parameter *θ*_c_ to denote the varying scattering information between them, including two situations. The calculation of the pseudo-scattering entropy parameter is performed [33]:

(4) $$H_{c}=-\sum_{I=1}^{2}p_{i}{log}_{2}p_{i};0\leq H_{c}\leq 1$$

where: $p_{1}=\frac{1}{1+q}$ and $p_{2}=\frac{q}{1+q}$ are the two pseudo - probability measures with *p*_1_ ≥ *p*_2_. *H*_c_ = 1 for *p*_1_ = *p*_2_ (i.e., *q* = 1), whereas *H*_c_ = 0 for *p*_1_ = *p*_2_ (i.e., *q* = 0).

**Figure 5:**
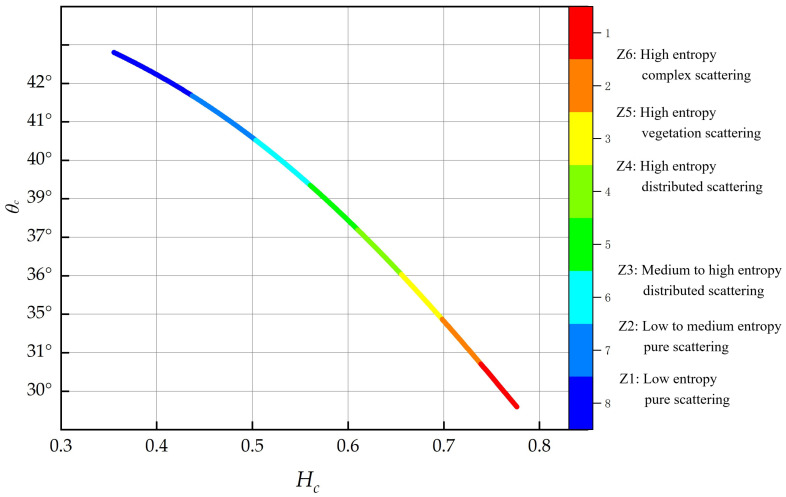
*H*_c_/*θ*_c_ two-dimensional clustering section. The curve is divided into six regions: Z1~Z6.

Figure 5 depicts the schematic flowchart of the unsupervised clustering system. The curve is the only viable clustering section of the *H*_c_/*θ*_c_ graph. In this study, six clustering zones are utilized based on the specific scattering characteristics of the target. These zones are referred to as Z1, Z2, Z3, Z4, Z5, and Z6. Additionally, *H*_c_ is divided into four subclasses: (0, 0.3), (0.3, 0.5), (0.5, 0.7), and (0.7, 1.0). Furthermore, *θ*_c_ is categorized into three subclasses: (0°, 15°), (15°, 30°), and (30°, 45°). Each of these zones corresponds to a distinct scattering phenomenon occurring in the picture [34].

To address the issue of the inability to use the *H*/α decomposition approach for characterizing the target scattering mechanism of GRD data. This work proposes the use of the 2 × 2 covariance matrix created by the scattering matrix and applies the Generalized Estimating Equations (GEE) to get the equivalent scattering angle (*θ*_c_). This angle is then utilized to characterize the target using GRD data. In addition, the *H*_c_/*θ*_c_ frame used in this work to create the clustering segments is obtained from the cross-polarization ratio *q*. However, while there is a link between the two quantities *q* and *θ*_c_, their physical interpretations exhibit diversity [35].

We performed plant monitoring with Sentinel-1A GRD SAR data within an unsupervised clustering framework, which exhibits superior adaptability compared to neural network models such as YOLO. SAR systems can consistently monitor vegetation under diverse meteorological situations, while neural network models such as YOLO, which depend on optical imagery, are more limited in unfavorable weather [36].

The crop growth dynamic monitoring system we offer is particularly well-suited for ongoing surveillance across extensive agricultural regions. SAR’s radar waves may infiltrate the vegetation canopy, acquiring data from both the surface and the interior of the vegetation, thus facilitating real-time monitoring of agricultural growth conditions, a task challenging for technologies such as YOLO that depend on optical imagery [37].

Furthermore, dual-polarization SAR data can proficiently differentiate surface features and precisely assess crop types and health problems. Neural networks such as YOLO excel in object detection and image classification, especially in tracking alterations in crop appearance and pest infestations; however, their dependence on optical sensors constrains their efficacy in adverse weather conditions, and they necessitate substantial labeled data for training to accurately identify crop growth stages [26]. An unsupervised clustering framework is more suitable for extensive, long-term crop monitoring in intricate weather circumstances. Nonetheless, YOLO is superior for real-time surveillance of particular crop manifestations and insect infestations.

The unsupervised clustering method can differentiate several scattering processes; nevertheless, the relationship between these mechanisms and particular stages of corn growth is still inadequately investigated. Furthermore, there is an absence of intuitive physical explanations and experimental data corroborating the response mechanisms of scattering characteristics at various growth stages.

The scattering characteristics of SAR data may be affected by ambient circumstances, leading to fluctuations and requiring additional experimental assessment of the model’s robustness. This work comprehensively examines the correlation between scattering mechanisms and various corn development phases utilizing an unsupervised clustering approach while corroborating the analysis with ground-measured data to improve the model’s precision in monitoring maize growth.

#### 2.5.2. Implementing Dual-Polarization Descriptors Based on Sentinel-1 in GEE

**Figure 6:**
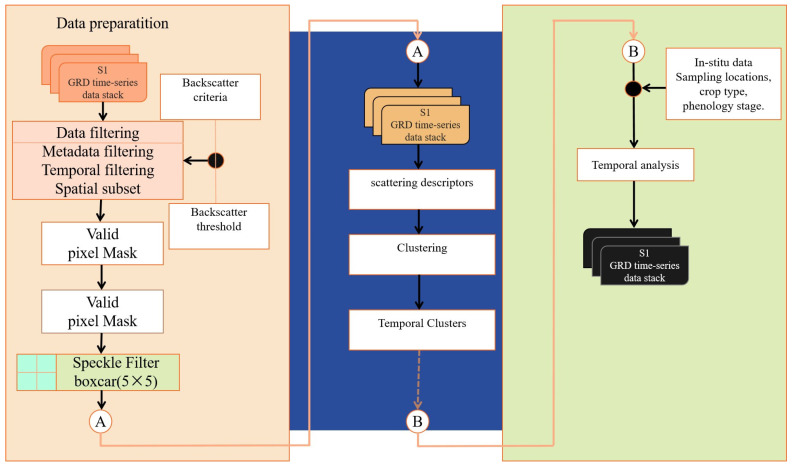
Dual-Polarization Descriptor from Sentinel-1 Dual-Polarization GRD SAR Data in GEE (“A” and “B” are process nodes in the flowchart).

Figure 6 illustrates the procedure for deriving requisite descriptors from Sentinel-1 dual-polarization GRD SAR data utilizing the GEE platform. Initially, we upload the backscatter coefficient data from GRD Sentinel-1 into the GEE platform, applying a mean filter to mitigate speckle noise and executing cloud filtering, encompassing metadata filtering, temporal filtering, and spatial boundary filtering. Furthermore, we produce a mask to establish a valid pixel data stack and transform the backscatter values into a linear scale. In the clustering step, we employ Formulas (2)–(5) to derive dual-polarization descriptors from the data stack, which are subsequently utilized to create *H*_c_/*θ*_c_ clusters for each scene inside the time stack.

We finalized the preparation of Sentinel-1A GRD SAR data on the GEE platform, encompassing noise reduction, geometric correction, and polarization decoding, and performed unsupervised clustering analysis utilizing this data. This approach obviates the necessity for dependence on local high-performance computing infrastructure. GEE, a high-performance cloud computing platform, offers robust computational resources that facilitate the effective processing of extensive remote sensing data. We secured the efficiency and stability of data processing by utilizing its integrated parallel computing and distributed storage technology. The unsupervised clustering analysis utilizing GEE for the experimental region measuring 4 km × 6 km required only 30 min to accomplish year-round monitoring of the complete growth cycle.

## 3. Results

### 3.1. Impact of Climate and Irrigation on Maize Growth

**Figure 7:**
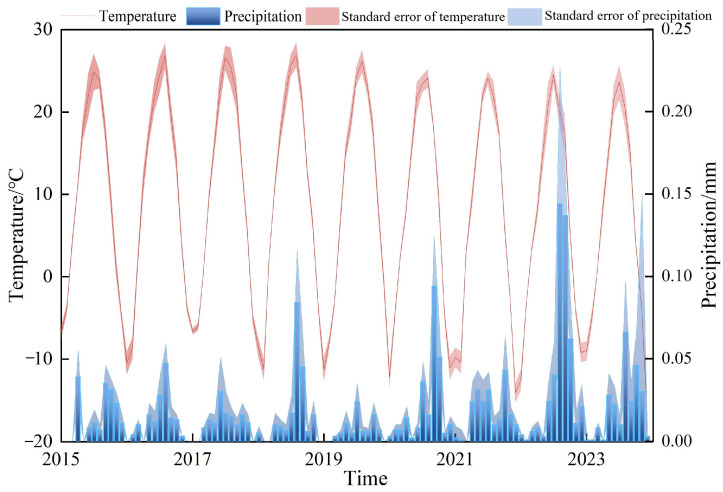
Average monthly temperature and total precipitation from 2015 to 2023.

We used ERA5 data from GEE and data from the Qinghai-Tibetan Plateau Science Data Center to extract cumulative monthly precipitation and mean monthly temperature from 2015 to 2023. This data allowed a comprehensive analysis of local meteorological impacts on maize’s life cycle. Over the 8-year period, the region exhibited a temperate continental monsoon climate, with most rainfall occurring in summer, coinciding with high temperatures (Figure 7).

High summer temperatures increased evaporation, creating dry conditions that accelerated maize growth but limited plant height. Between 2018 and 2023, annual precipitation reached its lowest levels. Rainfall peaked in June–July, and high temperatures occurred mainly in July–August. A slight decrease in daily average temperature helped reduce soil moisture evaporation and crop transpiration, delaying drought onset. Although heat resources declined during maize reproduction, light resources remained stable, improving temperature and rainfall conditions for crop development.

**Figure 8:**
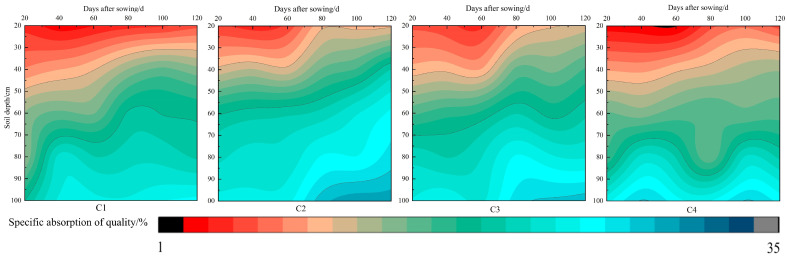
Dynamic changes of soil moisture content in 0–20 cm, 20–40 cm, 40–60 cm, 60–80 cm and 80–100 cm in 2020.

Soil moisture levels were monitored at depths of 0–20 cm, 20–40 cm, 40–60 cm, 60–80 cm, and 80–100 cm throughout maize growth (Table 2). Water absorption mainly occurred in the 20–60 cm layer (Figure 8), with primary roots concentrated between 0–40 cm. Soil moisture ranged from 12.54% to 17.69%. Before the elongation stage, water content in the 0–20 cm layer was low. The nodulation stage showed high drought resistance due to greater evaporation from the sandy loam surface than maize transpiration. After elongation, increased leaf area elevated transpiration, while irrigation and rainfall reduced topsoil evaporation, raising soil moisture.

Initially, maize roots focused on the 0–40 cm layer, limiting water uptake from deeper layers. However, as the plant grew, roots absorbed more water from 20–40 cm, leading to reduced soil moisture. During the filling stage, stable water levels resulted from cooler temperatures, lower surface evaporation, and decreased transpiration. Increased precipitation also maintained steady soil moisture, ensuring favorable growth conditions.

### 3.2. Impact of Evapotranspiration on Maize Growth

From April to September 2020, the average ET in the research area was 2342 mm. ET of maize strongly correlated with air temperature, with higher summer temperatures driving increased ET. A significant correlation between ET_0_; and temperature was observed (*r* = 0.67, *p* < 0.05), highlighting the importance of temperature in maize evapotranspiration. During July and August, the mid-growth stage, ET peaked at 9.3 mm/day, with monthly ET totaling 263 mm in this period and the highest value of 271.5 mm in July.

ET during the growth phase was notably higher than outside it, reflecting maize’s substantial water demand. The ET accumulation followed an S-shaped curve, starting low, peaking mid-growth, and decreasing as the crop matured. The mid-growth phase saw the highest ET due to increased leaf area, while in September, ET dropped as maize reached maturity. These results demonstrate that maize’s water needs vary across stages, underscoring the importance of stage-specific irrigation and water management strategies for optimal growth.

### 3.3. Analysis of Performance and Applicability Based on Bipolar Descriptors

We assessed the clustering framework of *m*_c_, *θ*_c_, and *H*_c_ by the analysis of 12 corn sample plots, examining the efficacy of descriptors in canopy morphological alterations. Initially, we examined the descriptors throughout the phenological stages in the time series. Subsequently, we conducted a performance assessment of the time series descriptors at 36 corn sampling locations. Ultimately, we examined the dynamic alterations in the *H*_c_/*θ*_c_ clusters.

**Figure 9:**
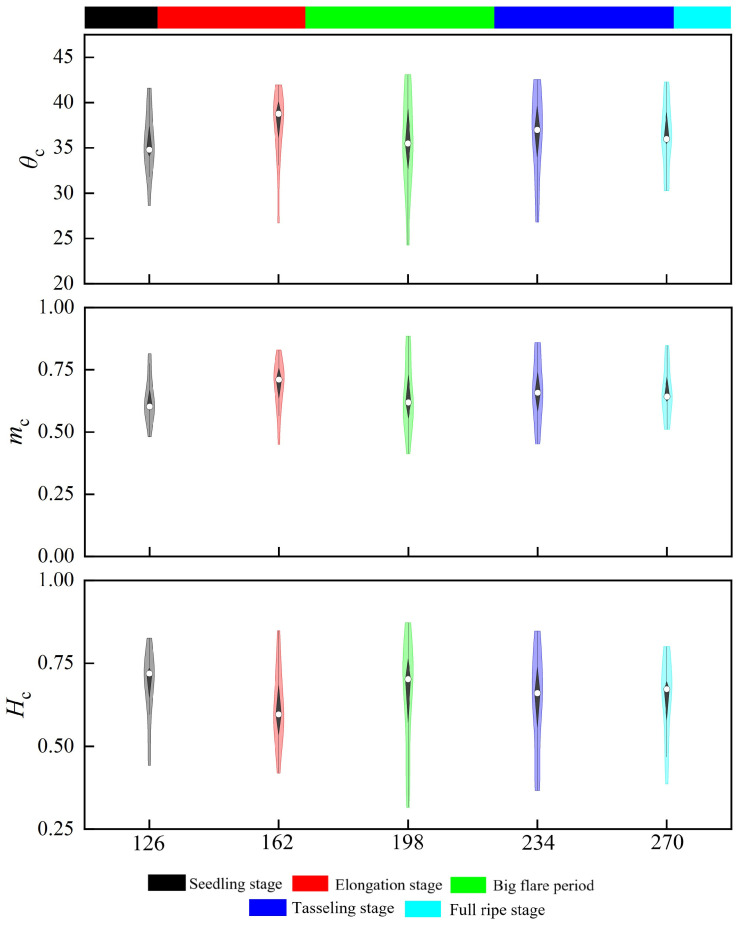
Temporal fluctuations of *m*_c_, *H*_c_, and *θ*_c_ at each fertility stage of maize. The white dots indicate the median values, while the black bar in the center shows a traditional box-and-line layout. Kernel density estimates are shown on both sides of the box-and-line plot to illustrate the distribution’s form. The sample size for each of the Seedling Stage, Elongation Stage, Big Flare Period, Tasseling Stage, and Full Ripe Stage is 16.

The time series of the clustering framework was examined utilizing violin plots, in which the breadth of the plot indicates the chance of selecting points assuming a specific value. The results indicate that over the time series, the three polarization descriptors are responsive to morphological alterations in the maize growth cycle (refer to Figure 9). The scattering characteristics during the seedling stage are affected by both soil and vegetation. Following the development of the canopy in the large bell mouth stage, the scattering properties undergo alterations, with elevated scattering values primarily emanating from the soil and partially from leaves and tillers, while approximately 13% of data points constitute tiny clusters (Z3). The scattering characteristics of specific polarization descriptors are elaborated in [23].

During the jointing stage, *H*_c_ diminishes, signifying a transition in the plant’s growth emphasis towards the development of reproductive organs. The increase in *m*_c_ and *θ*_c_ indicates heightened root activity and an augmented need for water.

During the extensive bell-mouth phase, *H*_c_ increases while *m*_c_ and *θ*_c_ diminish, signifying substantial variations in descriptor distribution across several growth modes. Throughout this interval, canopy density escalates, scattering entropy amplifies, and data points migrate towards medium and high entropy regions, with 53.7% of data points aggregated in the medium entropy region and 33.3% in the high entropy region. During the tasting phase, *m*_c_ and *θ*_c_ are often stable, signifying that maize has progressed into the advanced nutritional stage. The unpredictability of radar scattering leads to increased variability in scattering properties, resulting in a notable decrease in the standard deviation of the sample distribution while data clusters transition from the medium entropy zone to the high entropy zone. This alteration pertains to the structural modifications in the canopy during the tasseling phase, as biomass progressively reallocates to the upper canopy, with scattering predominantly originating from this region and the majority of data points concentrating in the high entropy zone (Z5) [21].

**Figure 10:**
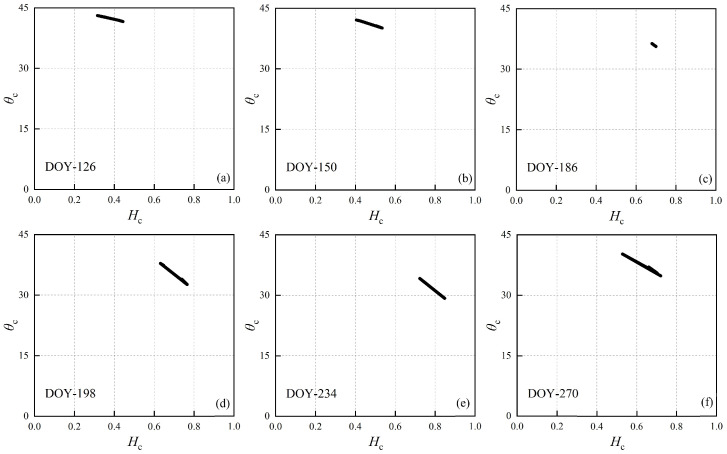
Clustering of maize full life span data. (**a**–**f**) represents the time varying *H*_c_ in the growth cycle.

During the maturity stage, canopy moisture content decreases swiftly, and the SAR signal’s penetration into the canopy intensifies, resulting in a greater contribution from the surface to backscatter. The distribution spread of *m*_c_ and *θ*_c_ diminishes, signifying enhanced homogeneity in the scattering mechanisms. The decrease in scattering entropy results in the *H*_c_/*θ*_c_ clusters transitioning from the random scattering region to the high entropy zone (Z6) (refer to Figure 10).

The results indicate that scattering mechanisms display significant sensitivity during the corn growth cycle, accompanied by a stable transition. We delineated the unique scattering mechanisms at several corn growth stages utilizing dual-polarization descriptors and a clustering methodology.

### 3.4. Analysis of the Dynamic Growth of Maize with Dual Polarization Descriptors

**Figure 11:**
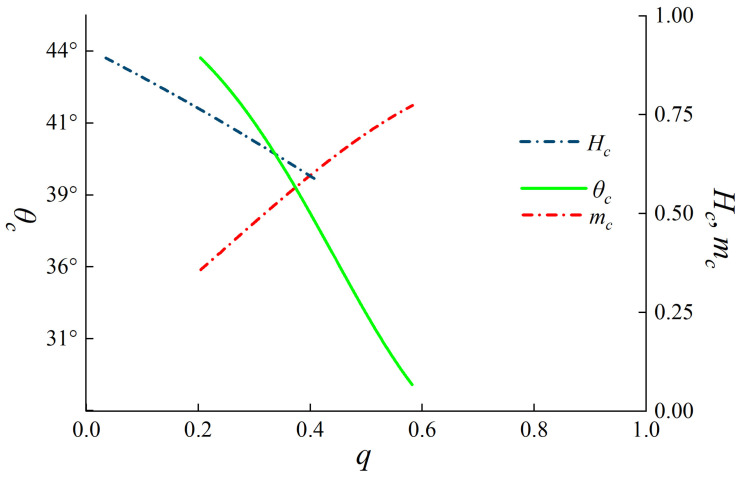
Proposed descriptors (*θ_c_*, *m_c_*, *H_c_,* and *q*).

**Figure 12:**
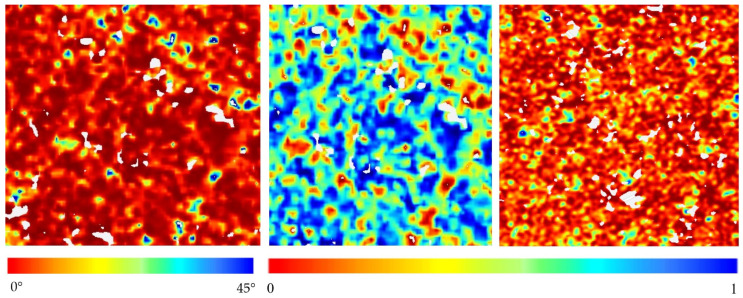
Pseudoscattering type parameter (*θ*_c_), co-pol purity measurement time change (*m*_c_), and pseudo scattering entropy parameter (*H*_c_).

Figure 11 and Figure 12 show the changes in *m*_c_, *θ*_c_, and *H*_c_ with time and space throughout the growth stages of maize in the research region. The fluctuation of these three characteristics was noticeable in most fields as the crop progressed from early leaf development to maturity.

On day 153, the majority of the maize *m*_c_ values were rather high, with an average of 0.64 ± 0.07. The *θ*_c_ value was measured at 36.18° ± 3.01°, while *H*_c_ was at a moderate to high level of 0.67 ± 0.24. The primary reason for this was the extensive coverage of maize in the area, which hindered major nutrient growth and leaf development. Therefore, the scattering reaction is determined by the qualities of the soil, such as moisture and surface roughness. The impact of soil roughness on the backscatter response was substantial, leading to the aggregation of sample pixels in the low to medium entropy pure scattering zone (referred to as Z1 and Z2) in *H*_c_/*θ*_c_. There was a consistent pattern of declining moisture content values as the crop development progressed into the male withdrawal stage. Therefore, on day 210 of the year, the *m*_c_ values were measured to be at medium to low levels, with a mean value of 0.51 and a standard deviation of 0.11. The density of the canopy rose as the crop biomass grew from the male pumping stage to maturity. Furthermore, the scatter reaction was mostly influenced by the top canopy. The maize had a *θ*_c_ value of 36.96° ± 1.31°, which suggests that there was little random scattering inside the resolution cell. The *H*_c_ value of maize, which is influenced by its randomly aligned canopy structure, was found to be 0.65 ± 0.05. Furthermore, there was a substantial growing trend seen.

The random structure of the DOY-210 vegetation made maize a notable feature in the high-entropy vegetation dispersion zone (Z5). The three polarization descriptors exhibited a reversal trend during the first phases of maize senescence, mostly caused by a reduction in canopy moisture resulting from changes in morphological characteristics.

Figure 11 illustrates that the moisture content (*m*_c_) of maize was 0.63 ± 0.07 at maturity (DOY-246), indicating a significant rise. Currently, the patterns of *θ*_c_ and *H*_c_ in Figure 12 are comparable. Nevertheless, the enhanced capacity of radar waves to penetrate the moderately arid maize canopy results in notable alterations in polarization characteristics. This suggests that a decrease in the amount of water in the vegetation leads to a decrease in the weakening of the SAR signal within the area being observed, resulting in an improved ability to penetrate through the maize plants.

This work used GRD SAR data to particularly examine the phenological phases of maize by analyzing the physical scattering features of the target electromagnetic waves. The temporal study revealed that the descriptors exhibited a significant level of sensitivity at various phases of maize phenological development. The ranges of *θ*_c_, *m*_c_, and *H*_c_ varied from 26.82° to 42.13°, 0.48 to 0.89, and 0.32 to 0.85, respectively, from the nodulation stage to the maturity stage of maize. Furthermore, the clustering system successfully captured the distinct phenological phases of maize. During the phases of maize leaf growth and nodulation, 80% of the sample sites were concentrated in areas with low to medium entropy and pure scattering. During the male extraction development stage, all sample stations were relocated to areas with high-entropy vegetative dispersion. Later, during the maturity stage, the sampling sites were grouped into high-entropy vegetation and high-entropy distribution scattering zones. These zones accounted for 65% of the sample points in the high-entropy distribution scattering zones.

## 4. Discussion

### 4.1. The Importance of Bipolar Descriptors in Combination with Unsupervised Clustering Frameworks

This research effectively integrated dual-polarization descriptors with an unsupervised clustering framework, yielding a robust time-series monitoring solution for dynamic corn growth. We employed dual-polarization descriptors to categorize the study region into six distinct scattering zones for the development of a dynamic monitoring model [36]. Dual-polarization descriptors provide an enhanced study of the physical attributes and growing conditions of crops and serve as an efficient data pretreatment method prior to implementing the unsupervised clustering framework [37].

The findings indicate that dual-polarization descriptors offer enhanced polarization data (including HH and VV), successfully differentiating various crop growth phases. Mandal et al. utilized DpRVI SAR data to introduce a novel vegetation index for evaluating the dynamic growth indicators of canola, soybean, and wheat, effectively extracting three physical factors of canola growth with correlation coefficients *R*^2^ between 0.75 and 0.82 [38]. Following the implementation of the unsupervised clustering framework, performance was enhanced by 7.21% to 9.33% relative to this method.

Mandal et al. retrieved biophysical indicators, including the vegetation area index and vegetation water content index, utilizing compact-polarization SAR data. Linear regression analysis revealed correlation coefficients for wheat and soybean ranging from 0.62 to 0.85 [6]. Rocío Ballesteros et al. used satellite platforms (Landsat 5TM, 7 ETM+, 8 OLI, and Sentinel 2A MSI) and a multiple linear regression model to monitor maize growth, demonstrating high accuracy with an R^2^ of 0.779. In comparison, the use of dual-polarization technology improved performance by 7.32% [39]. The dual-polarization descriptors introduced in this study enhance the extraction of biophysical data, yielding superior performance improvements and stability. By amalgamating data from two polarization channels, dual-polarization descriptors diminish data redundancy and enhance data accuracy. Zhang et al. indicated that dual-polarization descriptors exhibit sensitivity to minor alterations in surface characteristics, providing a superior advantage in detecting dynamic changes across several crop growth stages in comparison to vegetation indices and other biophysical factors [40]. SAR data can penetrate clouds and vegetation, facilitating data collection in overcast and rainy circumstances, thereby providing all-weather surveillance [41].

Dual-polarization SAR data possesses superior resolution, facilitating soil moisture assessment and aiding in the evaluation of crop growth conditions [42]. In the chosen study region, dual-polarization descriptors yield more comprehensive time-series data and, via the unsupervised clustering framework, may proficiently identify alterations in scattering mechanisms throughout crop development. Compared to the YOLO deep learning technique, dual-polarization descriptors provide benefits in time-series monitoring, spatial resolution, and independence from labeled data, rendering them appropriate for extensive surface change monitoring and crop dynamics research [26]. Table 3 illustrates the distinctions and benefits of dual-polarization descriptors in relation to the YOLO deep learning method for plant monitoring.

It is important to acknowledge that, although dual-polarization descriptors well capture crop growth patterns, dual-polarization data is vulnerable to noise, especially in settings of low signal-to-noise ratio [43]. Moreover, dual-polarization data typically possesses a greater size than single-polarization data, necessitating increased processing resources, hence prolonging the modeling duration [1]. The duration of modeling escalates with the expansion of the study area and the length of the time series [44]. Nevertheless, the dual-polarization metrics and the unsupervised clustering framework introduced in this study exhibit exceptional efficacy in monitoring dynamic maize growth, offering a valuable reference for sustainable agricultural advancement.

### 4.2. Insights Gained from Dual Polarization Descriptors in the Monitoring of Physical Parameters in Maize

This research utilizes C-band Sentinel-1 dual-polarization GRD SAR data to assess the phenological stages of maize in Dengkou County inside the Hetao Irrigation District, employing a clustering framework for dual-polarization descriptors. Time-series data of maize were obtained, concentrating on the analysis of fluctuations in three polarization descriptors—*m*_c_, *θ*_c_, and *H*_c_. These parameters demonstrated substantial variations across the majority of fields from the seedling stage to maturity, facilitating a more accurate evaluation of crop growth circumstances [45].

The research indicates that the variation in *θ*_c_ is predominantly affected by the upper layer of the crop canopy, with the backscatter response exhibiting heightened sensitivity to structural alterations within the canopy [46]. The *m*_c_ descriptor is intricately linked to variations in canopy density and is substantially correlated with biomass production. [47]. The ranges of variation for the three parameters are as follows: *θ*_c_ spans from 26.82° to 42.13°, *m*_c_ extends from 0.48 to 0.89, and *H*_c_ varies from 0.32 to 0.85.

During the growth cycle, crop development was robust, resulting in a canopy structure that became increasingly uniform, simplistic, and ordered, accompanied by a steady reduction in scattering angle [48]. Sonobe et al. observed that these three polarization descriptors had a reversal in trend at the later phases of crop growth (i.e., early senescence), primarily attributable to random alterations in morphological characteristics resulting in less canopy moisture [49]. This corresponds with the results of Wali et al., which demonstrate that as crops develop, the radar’s capacity to penetrate the desiccated canopy enhances, resulting in notable alterations in backscatter properties.

SAR signals in the C-band exhibit heightened sensitivity to moisture, and variations in crop water content can considerably influence SAR signal attenuation, particularly inside the resolution cell [50]. The crop canopy produces significant reflections, especially during periods of thick growth, where numerous scattering effects amplify the backscatter signal. As crop biomass rises, particularly during vigorous development phases such as heading and grain-filling, crop volume expands and structure becomes more intricate, leading to increased scattering inside the crop and further amplifying backscatter intensity [51]. This phenomenon is particularly evident in dual-polarization channels.

The VV channel is responsive to the vertical architecture of crops (e.g., stems) and can indicate variations in canopy height and density [52]. As crop biomass escalates, the backscatter intensity in the VV channel correspondingly climbs. The VH channel is more responsive to random scattering inside the canopy, with its scattering intensity markedly increasing at elevated biomass stages while exhibiting minimal response in low-biomass crops [53]. The HH channel primarily captures horizontal structural dispersion, including the arrangement and width of leaves. During periods of elevated crop biomass, the background intensity in the HH channel indicates the relative variations between leaves and stems.

Furthermore, the DpRVI index, by incorporating backscatter from the VV and VH channels, is especially effective for evaluating canopy structure in medium- to high-biomass crops [54]. The polarization ratio indicates the scattering variations among distinct polarization channels and is generally associated with crop biomass and structural intricacy. High-biomass crops, such as maize and soybeans, demonstrate an elevated polarization ratio, indicating increased random and multiple scattering effects [55].

These polarization characteristics offer enhanced insights for monitoring crop growth. The three polarization descriptors derived from dual-polarization Sentinel-1 GRD SAR data exhibit significant sensitivity in assessing maize growth phases [56]. This work employed the GEE platform to obtain and analyze high-frequency time series data from Sentinel-1, facilitating effective geographical analysis of agricultural phenological stages and offering robust research recommendations for crop development and ecosystem monitoring.

### 4.3. Limitations and Prospects

The primary aim of this study is to observe the dynamic alterations of maize across several growth stages, particularly over extended time periods, with an emphasis on crop growth conditions. The proposed method inadequately considers the influence of environmental conditions on maize growth and the variations in scattering characteristics resulting from structural changes in polarization parameters over distinct crop growth cycles [57]. This approach is more appropriate for crops that exhibit substantial alterations in stem and leaf morphology throughout growth, hence imposing specific constraints regarding crop varieties. Moreover, particular concerns about crops must be addressed, particularly when moisture content is inconsistent, as polarization metrics may not reliably indicate crop growth traits [52]. In areas with regular precipitation, saturated soil exhibits a greater dielectric constant, which can reflect an increased amount of microwave signals, resulting in enhanced backscatter, hence elevating noise levels in the data and diminishing analytical accuracy.

Precipitation can elevate the moisture levels on crop leaves and stems, augmenting SAR signal reflection within the canopy, especially in the VV channel, potentially leading to discrepancies in growth parameters [58]. The influence of wind speed on SAR signals is primarily evident in the tilting of crops, which can modify the numerous scattering patterns between the canopy and the soil, particularly in the HH and VV polarization channels, hence intensifying variations in backscatter strength [59]. Temperature does not directly influence SAR signal propagation; however, it can indirectly alter the backscatter signal by affecting the crop’s physiological condition and environmental moisture levels. The data quality and acquisition frequency of Sentinel-1A may change across various locations. In certain regions, data frequency may be diminished or gaps may exist, thereby impacting the comprehensive monitoring of the entire crop growth cycle [60].

Future studies may enhance this strategy by performing field validations across diverse crops to assess the model’s correctness, thereby more effectively representing the growth status of varied crops. Furthermore, it is advisable to utilize multivariate methods by integrating other climatic indicators and soil type variables as inputs for the algorithm to further investigate and resolve critical driving elements that the model has not thoroughly analyzed. Research indicates that the integration of these variables can markedly enhance predictive accuracy in agricultural settings [61].

Furthermore, implementing explainable methodologies to augment the model’s interpretability is a viable opportunity for enhancement. This study is significantly dependent on Sentinel-1 GRD SAR data, potentially impacting the accuracy of the findings. A future study should integrate data from the NASA-ISRO Synthetic Aperture Radar Mission (NISAR) and the Sentinel SAR network to enhance the accuracy and scope of the analysis.

The maize dynamic monitoring method we proposed can be refined by employing customized algorithms for crops in various regions to enhance the model’s accuracy and generalizability, thereby offering a more effective instrument for global agricultural phenology monitoring. These enhancements and expansions will further optimize the efficacy of our technology in monitoring the dynamic growth of maize.

## 5. Conclusions

This study, based on Sentinel-1 dual-polarization GRD SAR data, introduces three polarization descriptors: *θ*_c_, *m*_c_, and *H*_c_. Additionally, we designed an unsupervised clustering framework for analyzing the maize growth in the research area. By combining dual-polarization descriptors with the clustering framework, we analyzed the temporal dynamics of maize growth at the Dengkou Experimental Station in the Hetao Irrigation District.

The results indicate that this method is highly sensitive in assessing the dynamic growth performance and accuracy of maize at different phenological stages. (1) Based on the polarization descriptors and unsupervised clustering, we divided the maize growth process into six different scattering regions, each representing a different physical scattering mechanism. During the leaf development stage, approximately 80% of the maize sampling points were concentrated in the low to medium entropy scattering region. At the tasseling stage, clusters gradually shifted to the high entropy vegetation scattering zone. In the maturity stage, over 60% of the sampling points were located in the high entropy distribution scattering zone. (2) The parameters *θ*_c_, *m*_c_, and *H*_c_ exhibited significant variations across different phenological stages, with specific ranges of 26.82° to 42.13°, 0.48 to 0.89, and 0.32 to 0.85, respectively. As the maize growth cycle progressed, the maize structure became more uniform, simple, and orderly, with a gradual decrease in scattering angles, reflecting the physiological changes of the crop from development to maturity. (3) To further improve the accuracy of growth stage identification, we used a genetic algorithm to generate regional distribution maps of maize phenological stages, making crop growth condition assessments more precise.

The monitoring based on Sentinel-1A GRD SAR data provides a novel perspective and tool for dynamic monitoring of crop growth and prediction of agricultural yields. This study offers new insights into increasing agricultural production and promoting sustainable development, laying the foundation for future crop growth monitoring and yield prediction, and providing a reliable scientific tool for these efforts.

## Figures and Tables

**Table 1:** Sentinel-1A Data Details and Specifications.

Date	DOY	Acquisition Mode	Incidence Angle Range (deg.)	Orbit
6 May 2019	126	IW	41.95–43.10	Ascending
18 May 2019	138	IW	38.94–41.91	Ascending
30 May 2019	150	IW	40.12–42.13	Ascending
11 June 2019	162	IW	38.99–40.64	Ascending
23 June 2019	174	IW	34.80–39.32	Ascending
5 July 2019	186	IW	35.61–36.13	Ascending
17 July 2019	198	IW	32.63–37.86	Ascending
29 July 2019	210	IW	26.73–32.04	Ascending
10 August 2019	222	IW	26.82–33.07	Ascending
22 August 2019	234	IW	24.26–34.41	Ascending
3 September 2019	246	IW	34.82–37.81	Ascending
15 September 2019	258	IW	35.92–38.77	Ascending
27 September 2019	270	IW	35.50–40.24	Ascending

**Table 2:** Soil field water holding capacity, saturated water content, and soil bulk weight.

Soil Depth (cm)	Field Water Retention (%)	Percentage of Saturated Water Content (%)	Volume Weight of Soil (g·cm^−3^)
0~20	21.14	36.29	1.55
20~40	21.49	36.43	1.56
40~60	21.67	36.89	1.56
60~80	21.88	37.42	1.48
80~100	21.83	37.51	1.38

**Table 3:** Dual polarization Sentinel-1A GRD SAR unsupervised clustering compared with YOLO algorithm.

Characteristics/Method	Dual-Polarization Sentinel-1A GRD SAR Unsupervised Clustering	YOLO Algorithm
Application Domain	Remote sensing, crop growth monitoring	Vegetation monitoring, computer vision
Input Data Type	SAR imagery (VV and VH polarizations)	Optical remote sensing imagery
Algorithm Type	Unsupervised learning, mainly for data clustering and time-series analysis	Supervised learning, CNN for classification and detection
Suitable Scenarios	Monitoring large geographic areas and dynamic changes (e.g., farmland)	Object detection in single-frame or real-time scenarios (e.g., autonomous driving, surveillance)
Data Processing	Extracting spatiotemporal features through polarization descriptors (ratio, difference, intensity)	Learning features through convolutional networks for object identification in image regions
Spatial Coverage	Large geographic coverage, suitable for monitoring Earth’s surface dynamics	Local image detection is typically used for detecting objects in smaller areas.
Time-series Analysis	Supports long-term, multi-occasion monitoring (data from multiple time points)	Not suitable for time-series analysis, it focuses on single-frame images or consecutive video frames.
Annotation Requirement	Unsupervised learning, no need for large amounts of labeled data	Requires a large amount of labeled data for supervised learning and object detection
Computational Cost	Suitable for large-scale data processing using platforms such as GEE	High computational cost, especially for real-time detection on high-resolution images
Precision	1. Improved accuracy in time dimension through multi-temporal SAR data 2. High spatial resolution, suitable for large-scale monitoring	1. High accuracy in real-time object detection, especially in dynamic scenes 2. Label quality of training data significantly impacts accuracy

## Data Availability

The data presented in this study are available on request from the corresponding author. The data are not publicly available due to privacy.
